# Psychometric validation of the Physician Well-Being Index-Expanded (ePWBI) among physician educators in Hong Kong

**DOI:** 10.1080/07853890.2025.2532121

**Published:** 2025-07-16

**Authors:** Linda Chan, Paul Po Ling Chan, Xiaoai Shen, Emma Victoria Marianne Bilney, Tai Pong Lam, Julie Yun Chen, George L. Tipoe, Fraide A. Ganotice

**Affiliations:** a Department of Family Medicine and Primary Care, The University of Hong Kong, Hong Kong, China; b The Bau Institute of Medical and Health Sciences Education, The University of Hong Kong, Hong Kong, China; c Department of Family Medicine and Primary Care, The University of Hong Kong-Shenzhen Hospital, Shenzhen, Guangdong, China

**Keywords:** Physician Well-Being Index-Expanded, ePWBI, physician educators, distress, well-being, psychometrics, validation

## Abstract

**Introduction:**

Physician educators’ distress and well-being are of emerging concern in academic medicine. The Physician Well-Being Index-Expanded (ePWBI) is known for measuring physician distress and well-being, yet its psychometric properties in Asian contexts, including Hong Kong (HK), remain unexamined. This study evaluated the reliability and validity of the ePWBI in determining the distress and well-being of HK physician educators.

**Method:**

This cross-sectional validation study recruited 333 physician educators using convenience sampling at a HK medical school from October 2020 to January 2021, during the COVID-19 pandemic. Participants voluntarily completed the 9-item ePWBI and 5-item World Health Organization Well-Being Index (WHO-5) instruments in an online survey. Psychometric validation included within-network analyses (confirmatory factor analysis [CFA], one-way ANOVA, independent *t*-tests), and between-network analyses (ROC curves and correlational analyses with the WHO-5).

**Results:**

Using data from 333 physician educators, the ePWBI demonstrated excellent construct validity. CFA results indicated good data fit to the a priori model: Comparative Fit Index=0.99, Tucker–Lewis Index=0.99, Standardized Root Mean Square Residual=0.05, and Root Mean Square Error of Approximation=0.02 [90% CI 0.00–0.05]. Most factor loadings ranged from 0.36 to 0.69 and were statistically significant (*p*<0.05). Significant age differences in distress levels were found [*F*(4,328)=5.39, *p*<0.001], with younger educators (aged 20–39) experiencing greater distress. However, no gender differences were observed [*t*(328)=−1.16, *p*=0.247]. Between-network analyses revealed significant correlations between the ePWBI and WHO-5 scores (−0.09 to −0.42), along with satisfactory ROC results, indicating acceptable internal consistency and good discriminatory power.

**Conclusion:**

The ePWBI appears to be a valid and reliable instrument for assessing the distress and well-being of HK physician educators. It shows promise as a tool for identifying those at higher risk of distress who could benefit from early tailored interventions and in practice, it could thereby strengthen mental health support systems in academic medical institutions.

## KEY MESSAGES

- The ePWBI showed strong construct validity, while internal consistency and discriminant validity when compared to the WHO-5 were both acceptable, supporting its use in assessing distress and well-being among Hong Kong physician educators.
- Younger physician educators (20–39 years old) reported higher distress, suggesting early-career professionals may benefit from tailored institutional support.
- The study extends the use of the ePWBI to an Asian context, advancing its applicability across cultures. Its brevity plus psychometric strength make it a practical tool for monitoring physician educator distress and well-being.

## Introduction

Physician distress is a well-documented global concern [1–3], further intensified by the recent COVID-19 pandemic [4]. Physicians who hold dual roles in clinical practice and academia may be particularly vulnerable to stress due to the competing demands of these settings [5,6]. Despite the fact that decreased well-being of physician educators has previously been associated with poorer patient care and reduced teaching effectiveness [7–9], there remains a notable research gap regarding the specific factors influencing their psychological distress and well-being. This foundational gap highlights the broader lack of research in this area.

Academic physicians often struggle to balance clinical responsibilities and scholarly expectations, particularly regarding research productivity [10]. Their additional non-clinical duties—such as teaching medical students and building their academic or research portfolios—can compromise their well-being [5] and contribute to work-home conflict [6].

The literature also presents inconsistent findings regarding the influence of age on distress and well-being in

physician educators [4,11–13]. Most studies have reported greater depersonalization and poorer mental health among younger physician educators, likely due to the challenge of managing multiple roles early in their careers [4,11,14]. Younger physician educators may also face added pressure to establish themselves professionally, compounding their stress [11]. Conversely, other studies have found higher levels of burnout among older physician researchers (mean age >37 years) [12] or no significant age-related differences in emotional exhaustion and personal depersonalization [13]. These mixed findings suggest that not all younger physician educators experience distress equally; some may benefit from effective coping strategies or supportive work environments to help them navigate the challenges of their multifaceted roles.

Gender also appears to have a complex and mixed influence on physician well-being

[8,15]. Research suggests that emotional exhaustion is twice as prevalent in females as in males [9,16]. Similarly, female family physician educators reported greater burnout and emotional exhaustion when compared to their male counterparts [4,1,5]. This could be due to caregiving responsibilities [17] and the difficulty of balancing professional and personal obligations, thereby increasing their susceptibility to work-life conflict [14,18]. In contrast, no significant gender differences were observed in emergency medicine faculty [8]. These conflicting results suggest that contextual factors, such as workplace environment or job demands—may differently affect men and women [4,9,14,16–18].

Furthermore, most of these studies have been conducted in Western contexts, which may not fully reflect experiences in non-Western settings. Cultural differences can shape perceptions of distress, support-seeking behaviors, and workplace expectations. Therefore, investigating the relationships be

tween physician educators’ distress, well-being, and sociodemographic factors, such as age and gender in Asian contexts—particularly in Hong Kong (HK)—is both timely and warranted.

Burnout prevalence among physician educators varies widely by specialty. Whilst Kavanagh and Spiro reported that 69.6% of otolaryngology physician educators exhibited burnout symptoms [7], physician educators from emergency medicine [8], family medicine [4], and cardiology [9] reported high burnout/emotional exhaustion at 39.1, 66.5, and >25%, respectively. Pediatric nephrology faculty members reported a relatively low occurrence of burnout (16.3%) [19] and more residents had burnout and psychological problems than their faculty counterparts [20]. These differences may be due in part to the variety of tools used to assess distress, pointing to another important gap in the literature: the lack of standardized, validated instruments for this population.

Brady et al. [21] reviewed four commonly used scales for measuring physician well-being, burnout, or fulfillment to be adopted by healthcare systems monitoring the health of their physician workforces. With the caveat that Brady et al.’s review [21], along with most of the validation literature, was done in a Western context, it still offered an illuminating assessment of four instruments and their different areas of focus and capability. Among them, the 7-item Physician Well-Being Index (PWBI) offered all-round screening capacity for both work related well-being and general distress, achieving good concurrent and convergent validity with a low number of items. In contrast, other scales were more tightly focused on job fulfilment, or conversely, burnout at work [21].

The 7-item PWBI [22], a time-efficient and reliable measure of distress tailored to physicians [23], was extended by Dyrbye and colleagues to a 9-item Physician Well-Being Index–Expanded (ePWBI) instrument for measuring physician distress and well-being [24]. The ePWBI included items on meaning in work and work-life integration. These additions enhanced the tool’s sensitivity in identifying those with greater well-being, making the ePWBI a promising tool for use among physician educators.

Another commonly used measure is the World Health Organization Well-being Index (WHO-5) [25], a 5-item instrument assessing subjective well-being in the general population [26]. Prior research has supported its validity among HK medical educators [25]. However, that study included healthcare professionals from various disciplines involved in the training of medical students/trainees or doctors [25], whereas the current study focuses solely on *physicians* who teach medical students/trainees or doctors. Therefore, we designed this cross-sectional study to assess the between-network construct validity between the ePWBI and WHO-5 given that some level of validation for the latter already existed among healthcare professionals in HK [25].

Taken together, previous findings have shown that physician educators’ well-being is complex and influenced by a variety of factors, including their gender, age, and cultural background [4,9,11,14,16–1,8]. However, much of this work has been conducted in Western settings, leaving Asian perspectives underrepresented with limited region-specific data. Furthermore, inconsistencies in the instruments used to assess physician educators’ distress signal the need for a valid and comprehensive tool tailored to this population. Therefore, this study aimed to examine the psychometric properties of the 9-item ePWBI among physician educators in HK, marking the first validation study of its kind in Asia. Specifically, our investigation focused on the following objectives:

1. Assess the validity and reliability of the ePWBI in a HK physician educator population.
2. Explore associations between physician educators’ distress, age, and gender.
3. Evaluate between-network construct validity by correlating ePWBI scores with the validated WHO-5 Well-Being Index.

We also hypothesized the following:

Hypothesis 1: Younger physician educators would report experiencing higher levels of distress.

Hypothesis 2: Female physician educators would report experiencing higher levels of distress.

Hypothesis 3: ePWBI scores would demonstrate strong between-network validity through significant correlations with WHO-5 scores.

## Materials and methods

This was a cross-sectional validation study conducted from October 2020 to January 2021. A dual construct validation approach was adopted to examine the ePWBI in physician educators affiliated with the LKS Faculty of Medicine at The University of Hong Kong (HKU). This approach involved both within-network and between-network analyses to comprehensively assess the tool’s psychometric properties. The cross-sectional design allowed for the simultaneous collection of data at a single time point, enabling a robust analysis of the tool’s validity and reliability. Convenience sampling was used to invite physician educators engaged in teaching medical learners, trainees, and/or other physicians to participate in our research. Ethical approval was granted by the HKU Human Research Ethics Committee (ref: EA200136).

### Participants and sampling

Participants were physician educators affiliated with HKU’s LKS Faculty of Medicine, including both full-time academic and part-time honorary teaching staff. Eligibility criteria required participants to be actively involved in teaching medical students, trainees, and/or other physicians [25]. A convenience sampling strategy was used to recruit participants. A minimum sample size of 319 was determined based on calculations from a prior study on the well-being and resilience of HK nursing students (*r* = 0.378) [27], while a more conservative estimate of a small correlation (*r* = 0.2) was employed in our study.

Recruitment emails were sent to 1,902 HKU-affiliated medical educators *via* the university’s staff databases, which ensured eligibility by confirming employment status through official contracts and departmental vetting. Of the 435 medical educators who participated in the survey, 333 identified as physician educators based on their self-reported specialty and were included in the current study. As a gesture of appreciation, electronic coffee vouchers were offered to the first 150 respondents who completed the survey on a first-come, first-served basis.

### Approach to scale validation

Construct validation is a widely used method for assessing the psychometric properties of a scale and can be approached through both within-network and between-network validation [28,29]. Within-network validation focuses on the internal structure of the scale and was assessed through confirmatory factor analysis (CFA) to determine whether the data from HK physician educators fit the expected unidimensional model of the ePWBI. Internal consistency reliability was examined using Cronbach’s alpha and the factor correlation matrix was assessed. Between-network validation, as an external construct validation method, was conducted by analyzing the relationships between the ePWBI and other theoretically related constructs using correlation or regression analyses. In our case, we examined correlations between the ePWBI and the WHO-5, hypothesizing that these two instruments would be inversely related given their focus on distress and well-being, respectively. This dual approach—combining within- and between-network validation—provided a more comprehensive evaluation of the ePWBI’s construct validity. Few prior studies have adopted both approaches simultaneously, resulting in limited insight into the constructs being examined [25]. Consequently, our dual approach provided a more robust assessment of the ePWBI’s validity among HK physician educators.

### Study materials

An anonymous online questionnaire was developed in English using the Qualtrics platform (https://www.qualtrics.com/). The questionnaire included the ePWBI and WHO-5 instruments, as well as a series of sociodemographic questions, such as age, gender, ethnicity, relationship status, average hours worked per week, and the highest qualification attained in medical education specifically. Expert reviewers were consulted during the development phase of the questionnaire to ensure content and face validity. Additionally, their feedback enhanced the clarity of the questionnaire revisions by refining the wording of items and reducing redundancy. A small sample of physician educators also pilot tested the questionnaire to assess its overall usability and structure. This pilot phase led to several improvements, including fine-tuning the question flow and logical ordering of content. One key change based on pilot feedback was the standardization of all Likert-type response scales to maintain consistency in direction throughout the questionnaire, thereby enhancing user comprehension and response accuracy. The final version was distributed *via* email to eligible participants at the LKS Faculty of Medicine starting in October 2020, with the time period for returning the questionnaire ending in January 2021.

### Measurement tools

The Physician Well-Being Index-Expanded (ePWBI) [22–24] was the primary measure for our study. This 9-item self-report questionnaire assessed distress and quality of life. It was chosen over the original 7-item PWBI for its broader coverage of well-being dimensions, such as satisfaction with work-life integration [24], while maintaining the core focus on physician mental health and distress [23]. For the original seven items evaluating distress and the possibility of unfavorable personal or professional outcomes (e.g. ‘*Have you felt burned out from your work*?’), participants answered either YES or NO and were given 1 or 0 points, respectively [23]. Two additional items assessed meaning in work and satisfaction with work-life balance, which were used to identify individuals who were thriving [24]. Both questions were scored on a 7-point Likert scale (response option 1 or 2 = 1 point added to the score; response option 3 to 5 = 0 points gained; response option 6 or 7 = 1 point subtracted from the score). Previous studies reported that the scale had good internal validity, test-retest reliability, could be completed in <1 min, and had been widely used amongst physicians and medical trainees in Western settings [22–24]. While the ePWBI instrument is subject to copyright, interested readers can find more information on the following website (https://www.mywellbeingindex.org/research/) [22,23].

The World Health Organization Well-Being Index (WHO-5) [25,26], a 5-item self-report questionnaire was used to assess physician educators’ perception of their well-being over the prior two weeks at the time of completing the measure (e.g. ‘*I have felt calm and relaxed*’) [26]. All questions were scored on a 6-point Likert scale ranging from at no time (0) to all the time (5). Scores of the five items were summed and then converted to a final result where higher scores represented greater well-being (range 0–100). When screening for depression, a score of 50 or less suggested poor well-being. The scale had good construct validity [26] and had also been validated in HK [25]. The Cronbach’s alpha coefficient of the WHO-5 was 0.93 among HK medical educators, indicating excellent internal consistency [25]. Further details on the WHO-5 instrument questions and scoring method can be found in Appendix A.

### Data collection

To minimize participant burden, the ePWBI, WHO-5, and sociodemographic questions (e.g. age, gender, ethnicity) were administered as part of a larger survey study investigating the well-being and distress of HK medical educators [25]. Data were collected through a self-administered Qualtrics questionnaire, which most participants completed within 5–10 min. The study was conducted over a four-month period. The survey was distributed *via* email to all medical educators affiliated with the LKS Faculty of Medicine, HKU using institutional mailing lists. To enhance response rates, a maximum of three reminder emails were sent to non-respondents. Before accessing the questionnaire, participants were presented with an information sheet, and written informed consent was obtained electronically. Each respondent was assigned a randomly generated identifier by Qualtrics, which allowed for basic participant tracking while maintaining anonymity. No personal identifying information was collected unless participants voluntarily provided their email address to receive a coffee e-voucher as a token of appreciation. Beyond the Qualtrics-generated identifier, no other participant tracking methods were employed.

### Data analysis

The participant sample and ePWBI response characteristics were summarized using descriptive statistics. To examine within-network construct validity, CFA was performed to assess whether the one-factor ePWBI scale was applicable to our participants by using the diagonally weighted least squares (WLSMV) estimation approach, which was appropriate for ordinal data, such as Likert scales [30]. The Cronbach’s alpha coefficient was used to determine internal consistency and scale reliability, which were deemed satisfactory for psychological scales if Cronbach’s alpha lay between 0.7 and 0.9 [31]. We also examined corrected item-total correlations, which reflected the correlation between each individual item and the total scale score excluding that item. Items with a corrected item-total correlation value of below 0.3 were considered for removal, as they may not have contributed meaningfully to the overall construct being measured [32].

To evaluate age- and gender-related differences in ePWBI scores, we used one-way analysis of variance (ANOVA) for age group comparisons and independent sample *t*-tests for gender comparisons. A Scheffé *post-hoc* test was applied following significant ANOVA results to identify specific group differences. Due to a small sample of participants aged 20–29, we combined the 20–29 and 30–39 age groups to ensure an adequate sample size and to improve the statistical power plus robustness of the analysis. Gender-based comparisons were conducted both at the scale level (overall ePWBI score) and at the individual item level to examine potential gender-specific patterns of distress. However, item-level comparisons across multiple age groups were not performed to minimize the risk of Type I error due to multiple testing. *p*-Values of <0.05 in two-tailed tests were considered significant.

To evaluate between-network construct validity, Pearson correlation coefficients were calculated between the ePWBI and WHO-5 scores. Correlation strength was interpreted as weak (*r* < 0.20), moderate (*r* = 0.20–0.80), and strong (*r* > 0.80). The receiver operating characteristic (ROC) analysis was performed to analyze the area under the ROC curve (AUC), thereby assessing the ePWBI’s ability to discriminate between different levels of well-being as defined by WHO-5 scores. Cut-off values of 50 and 60 on the WHO-5 were used to categorize participants as having poor [33] or good well-being [34], respectively. An AUC value >0.70 indicated acceptable discriminatory power [35]. Participants with missing data were excluded from the analyses. All analyses were performed using IBM Statistical Package for the Social Sciences (version 27) and R (version 4.4.4).

## Results

This study investigated the psychometric properties of the ePWBI among HK physician educators, with the aim of assessing its validity and reliability, examining age-plus gender-related differences in distress, and evaluating between-network construct validity through correlations with the WHO-5 Well-Being Index. The ePWBI demonstrated strong construct validity, good internal consistency, and identified meaningful age-related differences in distress.

### Descriptive statistics

Descriptive analyses were used to summarize the demographic profile and response characteristics of the sample. Of 435 medical educators who responded to an email invitation to participate in our study, 333 (76.6%) were physician educators, and therefore, only this population of physician educators was included in the following analyses. We also conducted *post-hoc* power analyses and the results indicated that our study achieved adequate statistical power for the appropriateness of the sample size used [36,37]. Detailed parameters and results are provided in the Supplemental Online Material. As reported in Table 1, most of the 333 physician educators were male (71.5%), ethnically Chinese (94.6%), and married (76.3%), while the most common age range was 40–49 years (34.5%). More than half reported working 40–59 h per week (52.9%), and the most common qualification they had in medical education specifically was a bachelor’s degree (39.3%).

**Table 1.** Sociodemographic characteristics of study participants.

Participant characteristics (*N* = 333)	*n* (%)
Age in years	
20–29	6 (1.8%)
30–39	57 (17.1%)
40–49	115 (34.5%)
50–59	90 (27.0%)
≥60	63 (18.9%)
Prefer not to say	2 (0.6%)
Gender	
Male	238 (71.5%)
Female	92 (27.6%)
Prefer not to say	3 (0.9%)
Highest qualification in medical education	
None	64 (19.2%)
Certificate	12 (3.6%)
Diploma	2 (0.6%)
Bachelor	131 (39.3%)
Master or above	108 (32.4%)
Other	16 (4.8%)
Working hours per week	
≤19	11 (3.3%)
20–39	23 (6.9%)
40–59	176 (52.9%)
60–79	85 (25.5%)
≥80	38 (11.4%)
Relationship status	
Single	39 (11.7%)
Partnered	20 (6.0%)
Married	254 (76.3%)
Separated	2 (0.6%)
Divorced	8 (2.4%)
Widowed	3 (0.9%)
Prefer not to say	7 (2.1%)
Ethnicity	
Chinese	315 (94.6%)
Caucasian	6 (1.8%)
Mixed/multiple	2 (0.6%)
Others	6 (1.8%)
Prefer not to say	4 (1.2%)

Table 2 summarizes participant responses for each ePWBI item. The findings suggested that two-thirds of physician educators strongly believed their work was meaningful (68.8%). However, nearly half disclosed having experienced emotional difficulties (45.6%). Additionally, one-third of physician educators experienced burnout at work (37.2%) and expressed concerns that their work was hardening them emotionally (36.9%).

**Table 2.** Physician Well-Being Index-Expanded Scale items (*N* = 333).

ePWBI items	Yes *n (*%)
1. Have you felt burned out from your work?	124 (37.2%)
2. Have you worried that your work is hardening you emotionally?	123 (36.9%)
3. Have you often been bothered by feeling down, depressed, or hopeless?	64 (19.2%)
4. Have you fallen asleep while sitting inactive in a public place?	87 (26.1%)
5. Have you felt that all the things you had to do were piling up so high that you could not overcome them?	81 (24.3%)
6. Have you been bothered by emotional problems (such as feeling anxious, depressed, or irritable)?	152 (45.6%)
7. Has your physical health interfered with your ability to do your daily work at home and/or away from home?	50 (15.0%)
8. The work I do is meaningful to me.	
Very strongly agree/strongly agree (6–7)	229 (68.8%)
Agree/neither agree nor disagree/disagree (3–5)	93 (27.9%)
Very strongly disagree/strongly disagree (1–2)	11 (3.3%)
9. My work schedule leaves me enough time for my personal/family life.	
Strongly agree/agree (6–7)	150 (45.1%)
Neutral (3–5)	103 (30.9%)
Strongly disagree/disagree (1–2)	80 (24.0%)

*Note.* ePWBI: Physician Well-Being Index-Expanded.

### Construct validity and reliability of the ePWBI

#### Within-network construct validity

**Figure 1.**
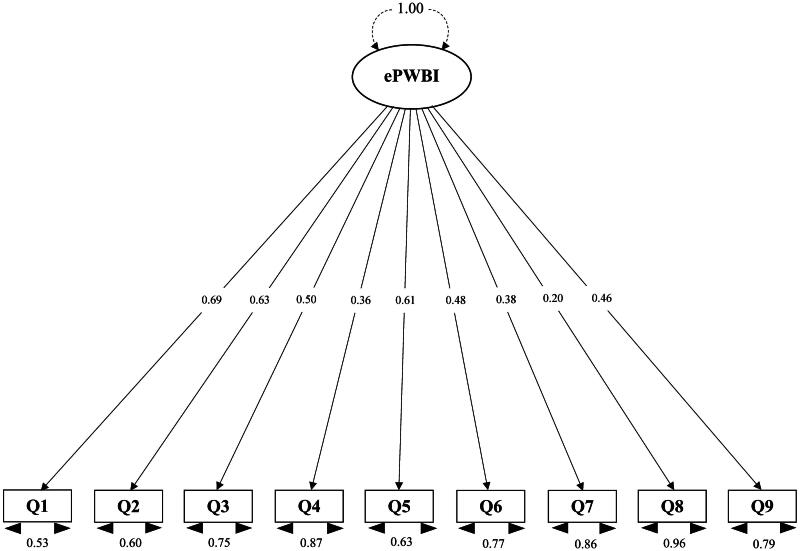
Confirmatory factor analysis of one-factor ePWBI model. *Note.* ePWBI: Physician Well-Being Index-Expanded Scale; Q1: Have you felt burned out from your work?; Q2: Have you worried that your work is hardening you emotionally?; Q3: Have you often been bothered by feeling down, depressed, or hopeless?; Q4: Have you fallen asleep while sitting inactive in a public place?; Q5: Have you felt that all the things you had to do were piling up so high that you could not overcome them?; Q6: Have you been bothered by emotional problems (such as feeling anxious, depressed, or irritable)?; Q7: Has your physical health interfered with your ability to do your daily work at home and/or away from home?; Q8: The work I do is meaningful to me.; Q9: My work schedule leaves me enough time for my personal/family life.

To evaluate the construct validity of the ePWBI, CFA was conducted. The one-factor model was assessed using maximum likelihood estimation to ensure its model fit with the collected sample data. The dataset satisfactorily met the necessary assumptions for CFA [38]. Comparative Fit Index (CFI) and Tucker-Lewis Index (TLI) values above 0.90, with Root Mean Square Error of Approximation (RMSEA) and Standardized Root Mean Square Residual (SRMR) values <0.08 [31] indicated acceptable model fit. The goodness-of-fit statistics are summarized in Table 3. Notably, the model demonstrated highly acceptable fit indices (CFI = 0.99, TLI = 0.99, SRMR = 0.05, and RMSEA = 0.02 [90% CI 0.00–0.05]), indicating a robust representation of the observed data by the ePWBI a priori model. Standardized factor loadings for each item on latent constructs and factor intercorrelations are illustrated in Figure 1. Most loadings fell within the range of 0.36–0.69 and were statistically significant (*p* < 0.05), indicating the reliability of observed variables in measuring their respective latent constructs. Although one item (#8) had a low factor loading (0.20), suggesting potential measurement inaccuracies, no significant modifications to the original CFA model were warranted based on modification indices and theoretical considerations. Overall, the model’s strong fit to the data substantiated the validity of the ePWBI model and affirmed its construct validity. To validate the scale further, we conducted analyses to assess the ePWBI’s reliability.

**Table 3.** Fit indices of the Physician Well-Being Index-Expanded Scale.

	*ꭕ*²(WLSMV)	*df*	*p*	CFI	TLI	SRMR	RMSEA (90% CI)
ePWBI	30.08	27.00	0.31	0.995	0.994	0.046	0.019 (0.000, 0.048)

*Note. ꭕ*^2^(WLSMV): weighted least squares mean and variance adjusted chi-square; *df*: degree of freedom; ePWBI: Physician Well-Being Index-Expanded Scale; CFI: comparative fit index; TLI: Tucker-Lewis index; SRMR: standardized root mean square residual; RMSEA: root mean square error of approximation.

#### Reliability

Internal consistency was evaluated using the Cronbach’s alpha coefficient. For the ePWBI, Cronbach’s alpha was 0.70 (see Table 4), indicating good internal consistency [31]. Corrected item-total correlations were examined to assess each item’s contribution to the overall scale. Most items met the ≥0.30 criterion, although item 8 fell below this threshold. Given the scale’s brevity, we additionally assessed mean inter-item correlation (*r* = 0.23), which was within the acceptable range of 0.20–0.40 [39], thereby supporting the reliability of the ePWBI. Inter-item Pearson correlations were also performed and displayed in Table 5. Most values fell within the range of 0.20 and 0.40, except item 8, indicating that most items were homogenous to a reasonable extent while possessing distinct variance. Item 8 correlated with other items, ranging from 0.05 to 0.17 in value. These results supported the overall reliability of the ePWBI scale.

**Table 4.** Independent *t*-tests and psychometric properties of the Physician Well-Being Index-Expanded.

	Total (*N* = 333)	Male (*n* = 238)	Female (*n* = 92)	*t*(328)	Cohen’s *d*	Cronbach’s α	Corrected item-total correlation
	M (SD)	M (SD)	M (SD)				
ePWBI	1.18 (2.49)	1.06 (2.52)	1.41 (2.41)	−1.16	2.49	0.70	
1. Have you felt burned out from your work?	0.37 (0.48)	0.34 (0.48)	0.42 (0.50)	−1.32	0.48		0.55
2. Have you worried that your work is hardening you emotionally?	0.37 (0.48)	0.38 (0.49)	0.34 (0.48)	0.70	0.48		0.51
3. Have you often been bothered by feeling down, depressed, or hopeless?	0.19 (0.40)	0.20 (0.40)	0.17 (0.38)	0.49	0.39		0.42
4. Have you fallen asleep while sitting inactive in a public place?	0.26 (0.44)	0.27 (0.44)	0.23 (0.42)	0.76	0.44		0.30
5. Have you felt that all the things you had to do were piling up so high that you could not overcome them?	0.24 (0.43)	0.22 (0.41)	0.30 (0.46)	−1.56	0.43		0.54
6. Have you been bothered by emotional problems (such as feeling anxious, depressed, or irritable)?	0.46 (0.50)	0.43 (0.50)	0.52 (0.50)	−1.53	0.50		0.38
7. Has your physical health interfered with your ability to do your daily work at home and/or away from home?	0.15 (0.36)	0.13 (0.34)	0.20 (0.40)	−1.39	0.36		0.32
8. The work I do is meaningful to me.	−0.65 (0.54)	−0.68 (0.52)	−0.61 (0.59)	−0.96	0.54		0.17
9. My work schedule leaves me enough time for my personal/family life.	−0.21 (0.81)	−0.23 (0.79)	−0.16 (0.86)	−0.69	0.81		0.38

*Note.* ePWBI: Physician Well-Being Index-Expanded; *M*: mean; *SD*: standard deviation.

**Table 5.** Correlation for the Physician Well-Being Index-Expanded Scale.

Item	Correlation coefficient (*r*^a^)								
	1	2	3	4	5	6	7	8	9
1. Have you felt burned out from your work?	–	0.48**	0.29**	0.28**	0.43**	0.27**	0.25**	0.08	0.36**
2. Have you worried that your work is hardening you emotionally?		–	0.34**	0.21**	0.29**	0.40**	0.20**	0.14**	0.23**
3. Have you often been bothered by feeling down, depressed, or hopeless?			–	0.18**	0.26**	0.35**	0.18**	0.11*	0.22**
4. Have you fallen asleep while sitting inactive in a public place?				–	0.22**	0.14**	0.21**	0.05	0.15**
5. Have you felt that all the things you had to do were piling up so high that you could not overcome them?					–	0.25**	0.31**	0.17**	0.40**
6. Have you been bothered by emotional problems (such as feeling anxious, depressed, or irritable)?						–	0.17**	0.12*	0.13*
7. Has your physical health interfered with your ability to do your daily work at home and/or away from home?							–	0.06	0.14**
8. The work I do is meaningful to me.								–	0.10
9. My work schedule leaves me enough time for my personal/family life.									–

*Note*. *p < 0.05, **p < 0.01.

^a^Pearson correlation test was performed.

### Associations with age and gender

#### Age differences (Hypothesis 1)

To evaluate age-related differences in physician educators’ distress, a one-way ANOVA was conducted across five age groups. Due to a small number of participants in the 20–29 age group, it was combined with the 30–39 age group to strengthen statistical power. Results revealed a significant effect of age on distress levels [*F*(4,328) = 5.39, *p* < 0.001]. *Post-hoc* Scheffé tests indicated that younger physician educators (20–39 years old; *M* = 2.35, *SD* = 2.63) reported significantly higher distress than their older counterparts in the 40–49 (*M* = 1.17, *SD* = 2.46), 50–59 (*M* = 0.81, *SD* = 2.15), and ≥60 (*M* = 0.52, *SD* = 2.52) age groups (see Table 6). These findings supported *Hypothesis 1*.

**Table 6.** Differences in Physician Well-Being Index-Expanded Scale scores across age groups.

Age groups	*n*	*M* (*SD*)	*F*	Scheffé *post-hoc* comparison
Group 1: 20–39	63	2.35 (2.63)	5.39**	2 < 1 3 < 1 4 < 1
Group 2: 40–49	115	1.17 (2.46)		
Group 3: 50–59	90	0.81 (2.15)		
Group 4: ≥60	63	0.52 (2.52)		

*Note. M*: mean; *SD*: standard deviation.

**p < 0.01.

#### Gender differences (Hypothesis 2)

Independent *t*-tests were conducted to explore gender differences in distress. No significant difference in overall ePWBI scores was observed between male (*M* = 1.06, *SD* = 2.52) and female (*M* = 1.41, *SD* = 2.41) participants [*t*(328) = −1.16, *p* = 0.247], thus not supporting *Hypothesis 2*. Item-level analyses similarly found no significant gender differences (see Table 4).

#### Between-network construct validity (Hypothesis 3)

To examine between-network construct validity, Pearson correlations were computed between ePWBI and WHO-5 scores. As shown in Table 7, ePWBI and WHO-5 score correlations ranged from −0.09 to −0.42, with most reaching statistical significance. These low-to-moderate negative correlations indicated that the ePWBI captured aspects of well-being that were distinct from the WHO-5. This finding supported the ePWBI’s between-network construct validity and thereby *Hypothesis 3*.

**Table 7.** Correlation for the Physician Well-Being Index-Expanded and World Health Organization Well-Being Index Scales.

Items	Correlation coefficient (*r*^a^)				
	1. I have felt cheerful and in good spirits.	2. I have felt calm and relaxed.	3. I have felt active and vigorous.	4. I woke up feeling fresh and rested.	5. My daily life has been filled with things that interest me.
1. Have you felt burned out from your work?	−0.30**	−0.39**	−0.34**	−0.40**	−0.29**
2. Have you worried that your work is hardening you emotionally?	−0.28**	−0.32**	−0.28**	−0.28**	−0.29**
3. Have you often been bothered by feeling down, depressed, or hopeless?	−0.31**	−0.39**	−0.25**	−0.34**	−0.32**
4. Have you fallen asleep while sitting inactive in a public place?	−0.11	−0.10	−0.09	−0.16**	−0.10
5. Have you felt that all the things you had to do were piling up so high that you could not overcome them?	−0.31**	−0.36**	−0.33**	−0.42**	−0.30**
6. Have you been bothered by emotional problems (such as feeling anxious, depressed, or irritable)?	−0.31**	−0.26**	−0.25**	−0.30**	−0.25**
7. Has your physical health interfered with your ability to do your daily work at home and/or away from home?	−0.16**	−0.14**	−0.14**	−0.21**	−0.16**
8. The work I do is meaningful to me.	−0.26**	−0.23**	−0.23**	−0.21**	−0.29**
9. My work schedule leaves me enough time for my personal/family life.	−0.27**	−0.30**	−0.29**	−0.39**	−0.23**

*Note*. **p < 0.01.

^a^Pearson correlation test was performed.

### Discriminant validity and ROC analysis

**Figure 2.**
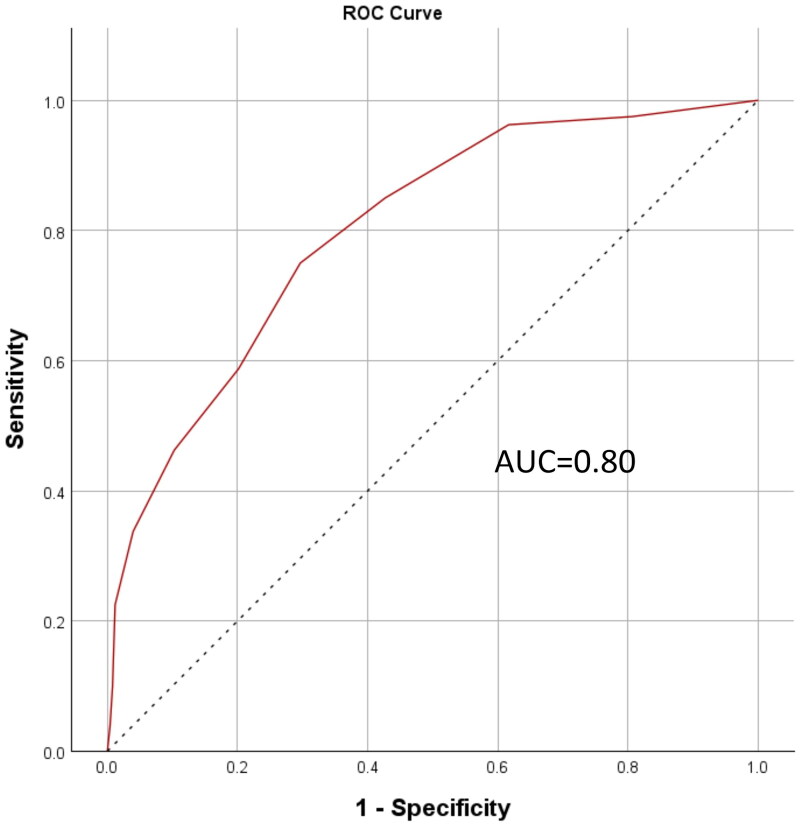
ROC curve (WHO-5 cut-off = 50). *Note.* ROC: receiver operating characteristic; AUC: area under ROC curve; WHO-5: 5-item World Health Organization Well-Being Index.

**Figure 3.**
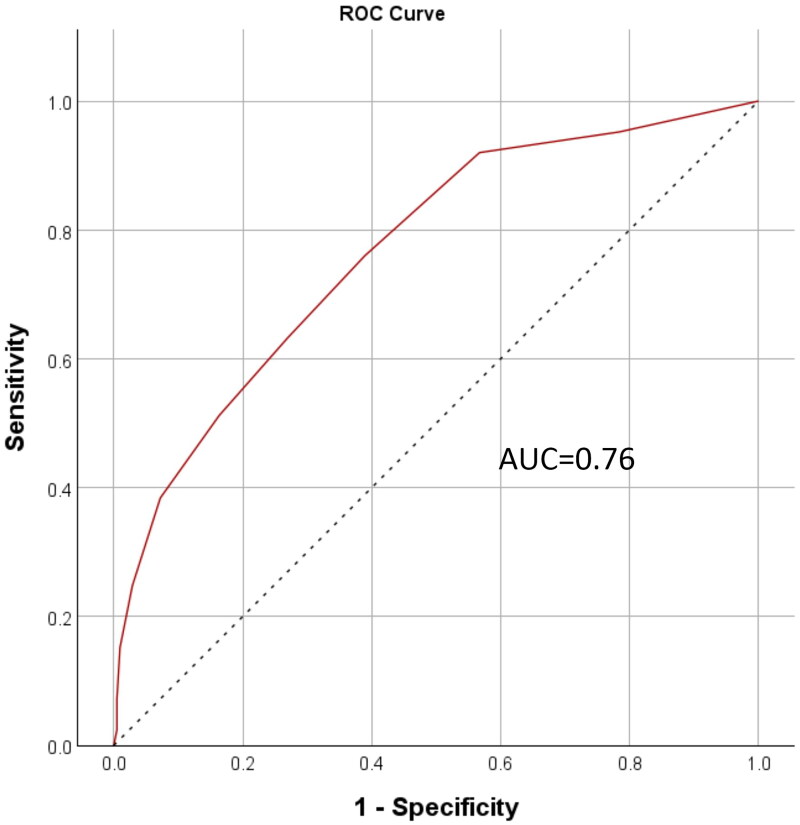
ROC curve (WHO-5 cut-off = 60). *Note.* ROC: receiver operating characteristic; AUC: area under ROC curve; WHO-5: 5-item World Health Organization Well-Being Index.

To assess the ePWBI’s ability to distinguish levels of well-being, ROC analysis was performed using WHO-5 cut-offs of 50 and 60. The AUC values were 0.80 (see Figure 2) and 0.76 (see Figure 3), respectively—both exceeding the 0.70 threshold for acceptable discriminatory power. These results highlighted the ePWBI’s utility in identifying physician educators with lower well-being.

In summary, the ePWBI demonstrated strong construct validity and acceptable internal consistency in this cohort. Distress levels among physician educators varied significantly by age group, with younger participants reporting higher distress, but no significant differences were observed by gender. The ePWBI showed acceptable discriminant validity when compared to the WHO-5, although convergent validity was limited.

## Discussion

To our knowledge, this study is the first to validate the ePWBI for assessing distress and well-being among physician educators in an Asian context and specifically in HK. Although the ePWBI has been widely utilized in North America across various disciplines [22–24,40], its cross-cultural applicability in Asian populations has remained under-explored. This limits the generalizability of the existing literature given the significant influence of societal and interpersonal dynamics on well-being [41]. By confirming the ePWBI’s strong construct validity, acceptable internal consistency, and good discriminant validity, our study offers valuable evidence that the scale is psychometrically sound for use in this context. Notably, the ePWBI effectively identified younger physician educators (aged 20–39) as a high-risk group for distress, supporting our first study hypothesis and highlighting a critical demographic requiring tailored support. These findings underscore the importance of using culturally sensitive and empirically validated tools to monitor well-being, particularly in academic medicine where early-career professionals may face unique stressors. In contrast, gender was not significantly associated with distress in this cohort, although this result should be interpreted cautiously given the sample’s gender imbalance. Our findings also lend support to the scale’s between-network construct validity, as shown by its meaningful, albeit modest, correlations with the WHO-5, further establishing its utility in well-being research.

### Cross-cultural comparison of psychometric properties

The within-network validity of the ePWBI was strongly supported by our CFA results, which confirmed a unidimensional latent structure with excellent model fit [38] (CFI and TFI >0.90; RMSEA and SRMR <0.08). These findings are consistent with previous North American validations of the PWBI, where the scale demonstrated robust psychometric properties and practical utility in identifying physicians at risk for low mental quality of life, fatigue, and suicidal ideation. For example, in a national U.S. sample of 6,994 physicians, a PWBI score of ≥4 was associated with a specificity of 81% and sensitivity of 73.3% for detecting significant distress [23]. Similarly, among residents, a threshold of ≥5 yielded 83.6% specificity for identifying those with poor mental quality of life or suicidal ideation [42].

Our results align with this body of work in demonstrating the ePWBI’s effectiveness in stratifying distress within an academic medicine cohort. However, our study also extends the utility of the tool by validating it in an Asian cultural context. This is a significant contribution given that well-being instruments often reflect assumptions rooted in Western individualism, which may not fully capture the relational and collectivist dynamics more prominent in Asian settings. For instance, item 8—related to thriving—showed a weaker factor loading in our CFA, which may reflect subtle cultural differences in how concepts like ‘thriving’ or emotional hardening are perceived in professional roles [43]. Despite this, item 8 retained theoretical relevance [44], and overall internal consistency remained acceptable [45], suggesting that the core construct of psychological distress remains consistent across cultures, though nuanced expressions may vary. Our findings indicate that while the ePWBI performs similarly to its North American counterpart in terms of psychometric strength, certain items may warrant further investigation or cultural adaptation to maximize interpretive accuracy and relevance in non-Western contexts.

### Age and gender differences

Consistent with our first hypothesis and corroborated by similar findings in Asian contexts, such as Japan [11] and Shanghai [46], younger physician educators (aged 20–39) reported significantly higher distress levels. This aligns with other studies noting that early-career

physicians often face intense pressure, limited autonomy, and uncertain career trajectories [4,11,14]. For example, Toyoshima and colleagues discussed that personal burnout among Japanese academic physicians in their study may be explained by their young age [11]. Moreover, a study by Wang et al. reported that the prevalence of burnout cases was significantly higher among young physicians under the age of 35 in Shanghai [46]. The identified differences between age groups illustrated the psychometric ability of the ePWBI to stratify the psychological distress of HK physician educators. These age-related findings underscore the need for tailored well-being interventions for younger faculty, potentially including mentorship, workload adjustment, or structured holistic wellness-related programs.

We found no statistically significant gender differences in distress which aligns with Lu et al.’s study of physician educators in emergency medicine [8] and a HK-based study using the WHO-5 to measure well-being among medical educators [25]. The results did not support our second hypothesis and contrast with other Western studies that have reported higher burnout rates among female physicians [4,9,16,17]. Several cultural and institutional factors unique to HK may help explain this divergence. For instance, women in HK’s higher education sector demonstrate higher rates of enrolment, grant submissions, and research funding success [47,48]. These patterns suggest a more equitable academic environment, potentially mitigating gender-related stressors, such as competition or systemic bias [49,50]. Such dynamics may contribute to reduced gender-related distress among female physician educators and could partially explain our findings. Additionally, the gender composition of our sample—predominantly male (71.5%)—could have limited our ability to fully examine gender effects, and subtle gender differences may have gone undetected. Furthermore, social norms around emotional disclosure and role expectations may influence how distress is reported across genders, particularly in professional settings [51,52]. These factors point to the need for more gender-balanced sampling in future studies, along with qualitative approaches that can uncover culturally specific expressions of distress and well-being. Exploring these intersectional dynamics in greater depth would enhance our understanding of physician educator well-being across different sociocultural contexts.

These findings highlight the ePWBI’s capacity to capture subgroup differences and the need for culturally informed, intersectional approaches to well-being research. Building on this, we next examined the scale’s between-network validity to assess its performance alongside an established well-being measure.

### Between-network construct validity

Our findings confirmed the ePWBI’s good discriminative power and acceptable between-network construct validity when compared to the WHO-5. Convergent validity remained modest, suggesting it measured a construct distinct from that assessed by the WHO-5. This is expected, as the ePWBI primarily measures psychological distress, while the WHO-5 focuses on positive well-being. The fact that both tools captured different dimensions of emotional health highlights the potential value of using them together for a more holistic assessment. Additionally, the AUC values, which surpassed the widely accepted threshold of 0.70, further supported the ePWBI’s good discriminative power, confirming its utility in identifying individuals experiencing distress. As such, our results suggest that the ePWBI may effectively evaluate multiple dimensions of psychological distress and well-being [23,24] in HK physician educators, giving support to both our third hypothesis and relevant previous literature [23,24,40,42]. Furthermore, it can be completed within a minute, which is highly suitable for the busy schedules of physician educators, making it a practical and effective tool for monitoring the various facets of physician educator distress and well-being [24]. These findings align with studies from North America that emphasize the practical utility of the ePWBI as both a research instrument and a frontline screening tool [24,53].

This study contributes to the growing literature on physician educator distress and well-being by providing initial evidence of the ePWBI’s construct validity among physician educators in HK, thereby extending its cross-cultural validity. Our findings support both within-network and between-network validity of the ePWBI, reinforcing its capacity to assess psychological distress and well-being in this academic medicine cohort. Notably, we identified younger physician educators (aged 20–39) as a group at higher risk of distress, while observing no significant gender differences. By validating the ePWBI in a HK context, this study fills an important gap in cross-cultural psychometric research in terms of understanding how such tools perform in an Asian setting. Moreover, it suggests that the tool holds promise for broader application across educational and healthcare contexts. These findings lay the groundwork for future studies to replicate and extend this validation across other regions, specialties, plus professional roles.

### Limitations

Our study had several limitations that should be considered when interpreting the findings:

- *First*, the reliance on self-reported data may have introduced social desirability bias, as participants may have provided responses they perceived as favorable rather than fully accurate. For example, physician educators may have underreported their levels of distress or overreported their sense of well-being. This tendency could be driven by a desire to present themselves as resilient, competent, or unaffected by stress, especially given the cultural emphasis on professionalism and emotional composure in medicine. This could have led to an underestimation of actual distress levels in the sample, thereby attenuating the strength of observed relationships and limiting the sensitivity of the ePWBI in identifying high-risk individuals. Future research could benefit from triangulating self-report data with qualitative interviews or supervisor assessments to provide a fuller picture of physician educator distress and mitigate potential biases.
- *Second*, the cultural homogeneity of our sample—predominantly Chinese physician educators in HK—limits the generalizability of the findings among non-Chinese populations. Cultural norms around mental health, emotional expression, and help-seeking behaviors vary significantly across societies, and these differences may influence how distress is reported and experienced. Furthermore, differences in societal attitudes toward distress, medical education systems, and healthcare system cultures across countries could shape stressors, coping mechanisms, and professional expectations. As such, the applicability of the ePWBI outside the HK context should be interpreted with caution. Although our study addresses a gap in cross-cultural validation, additional research is needed to assess the ePWBI’s psychometric performance in different Asian regions or areas outside of Asia to determine its global utility.
- *Third,* convenience sampling may have introduced selection bias. As participation was voluntary and recruitment was conducted via email using convenience sampling, it is possible that respondents who were more engaged, more distressed, or more interested in well-being were overrepresented. Additionally, individuals with time constraints or lower motivation may have been underrepresented. Future studies should consider probability sampling or purposive strategies to ensure more representative participation across the physician educator population.
- *Fourth*, variations in subgroup sample sizes—by gender and age—could have impacted the statistical power and stability of comparisons. The gender imbalance (with over 70% male participants) may have obscured potential gender-related effects. Ensuring a more balanced sample would improve the accuracy and generalizability of subgroup analyses.
- *Fifth*, while our study established within-network and between-network construct validity, we were unable to examine external validation of the ePWBI with constructs beyond well-being. Future validation studies should assess the ePWBI in relation to other relevant constructs, such as professional burnout, work engagement, or organizational support to further establish its robustness.
- *Finally*, the cross-sectional nature of the study offers only a single temporal snapshot, conducted during the fourth wave of the COVID-19 pandemic in HK. This period was marked by heightened clinical demands, rapid changes including a sudden pivot to online teaching, prolonged uncertainty, and social restrictions. As a result, the data may reflect context-specific stressors unique to the pandemic period, limiting its applicability during more typical conditions. Therefore, longitudinal studies are recommended to capture changes in well-being over time and provide a more comprehensive understanding of physician educators’ psychological trajectories under different conditions.

### Implications and future directions

Our study makes several contributions to the existing literature. First, it extends the validation of the ePWBI to an Asian context, addressing the critical need for culturally sensitive tools to monitor physician educator distress and well-being. The identification of young physician educators (20–39 years old) as a high-risk group for distress adds to the growing body of evidence on the vulnerability of early-career professionals in competitive academic environments, particularly within hierarchical and high-performance cultures, such as those found in HK. Second, this is the first study to directly compare the ePWBI and WHO-5 psychometric scales. By doing so, our research demonstrated the ePWBI’s discriminant validity relative to the WHO-5, highlighting its capacity to capture a broader spectrum of psychological distress and well-being dimensions. This adds support to the ePWBI as a distinct but complementary tool for well-being assessment, with unique relevance to occupational and psychological stressors.

Looking forward, future research could expand validation efforts to physician educators in other Asian regions and international contexts to assess the global utility of the ePWBI. Cultural norms, systemic structures, and attitudes toward distress can vary widely across countries, potentially affecting both the expression of distress and the measurement sensitivity of assessment tools. Therefore, further research could also refine or adapt the ePWBI to better capture such cultural nuances. Additional psychometric comparisons with other established distress and well-being scales could also strengthen the ePWBI’s standing as a reliable instrument across diverse professional and cultural environments.

Beyond cultural validation, further studies could incorporate more nuanced demographic and medical specialty variables. Additionally, intersectional influences could be explored, such as how age, gender, work experience, specialty, and teaching load interact with cultural or institutional dynamics to affect distress levels. For instance, younger female physician educators in male-dominated specialties may experience compounded pressures that single-variable analyses may overlook. Including such intersectional analyses could broaden the scope of future research and provide a richer understanding of physician distress, allowing institutions to tailor interventions more precisely.

Finally, longitudinal research is warranted to evaluate the ePWBI’s responsiveness over time and its capacity to track well-being trajectories, particularly in response to organizational changes, life events, or public health crises.

## Conclusion

Our study offers a novel contribution to the field by providing evidence supporting the validity of the ePWBI for assessing distress and well-being among physician educators in HK. Through strong psychometric indicators—including high CFA fit indices and acceptable internal consistency—the ePWBI demonstrated robust construct validity. Its ability to identify those aged 20–39 as a high-risk group for distress underscores its utility for detecting distress among physician educators. Additionally, the ePWBI’s discriminant validity relative to the WHO-5 highlights its value in capturing distinct dimensions of distress. These findings highlight the ePWBI’s potential as a culturally sensitive, time-efficient screening tool and importantly, enhances the range of mental health assessment tools available for physician educators, especially in Asian contexts. In settings like HK, where workplace competition and social expectations may heighten stress, use of the ePWBI for monitoring physician educator well-being could enhance mental health support systems within academic medical institutions and help identify those in need of early intervention plus support. Incorporating the ePWBI into regular faculty wellness assessments could also inform institutional strategies, guide resource allocation, and promote sustainable well-being practices among medical educators. Furthermore, our study lays a foundation for broader application by highlighting the ePWBI as a promising tool for cross-cultural validation and use in other Asian contexts or in global healthcare education systems. However, several limitations temper the generalizability of these findings. The use of convenience sampling may have favored physician educators who were more engaged or motivated, potentially introducing sampling bias. The cultural homogeneity of the sample (predominantly ethnically Chinese) and its gender imbalance (over 70% male) may have also shaped the observed patterns and warrant cautious interpretation. Further studies are therefore needed with more representative samples, which explore its application in other Asian settings, and compare its performance with additional validated psychometric instruments. Ultimately, beyond validating the ePWBI as a novel tool for assessing distress and well-being among HK physician educators, our study sets the stage for its broader validation and potential application in diverse cultural contexts globally.

## Supplementary Material

Supplemental online material.docx

## Data Availability

The dataset is available from the corresponding authors (Linda Chan and Fraide A. Ganotice) upon reasonable request.

## Appendix A

### The World Health Organization-Five Well-Being Index (WHO-5)

Please indicate for each of the five statements which is closest to how you have been feeling over the last two weeks. Notice that higher numbers mean better well-being.

Example. If you have felt cheerful and in good spirits more than half of the time during the last two weeks, select number three.

**Table:**

		All of the time	Most of the time	More than half of the time	Less than half of the time	Some of the time	At no time
1	I have felt cheerful and in good spirits	5	4	3	2	1	0
2	I have felt calm and relaxed	5	4	3	2	1	0
3	I have felt active and vigorous	5	4	3	2	1	0
4	I woke up feeling fresh and rested	5	4	3	2	1	0
5	My daily life has been filled with things that interest me	5	4	3	2	1	0

### Scoring

The raw score is calculated by totalling the scores on each of the five questions. The raw score ranges from 0 to 25, 0 representing the worst possible mental well-being and 25 representing the best possible mental well-being.

To get a percentage score ranging from 0 to 100, the raw score is multiplied by 4. A percentage score of 0 represents the worst possible mental well-being; a score of 100 represents the best possible mental well-being.

### Comment

A percentage score below 50 (or a raw score below 13) has been suggested as a cut-off for poor mental well-being and as an indication for further assessment for the possible presence of a mental health condition (e.g. depressive disorder).
