# Supplementary material for: Psychometric validation of the Physician Well-Being Index-Expanded (ePWBI) among physician educators in Hong Kong
Source: Ann Med. 2025 Jul 16;57(1):2532121. doi: 10.1080/07853890.2025.2532121 (PMC12269091; doi:10.1080/07853890.2025.2532121)
Supplement: Supplemental online material.docx [file IANN_A_2532121_SM1446.docx]

**Validation of the Physician Well-Being Index-Expanded (ePWBI) among Physician Educators in Hong Kong**

**Supplemental online material**

1. **Supplementary item 1. Parameters and Results of Post-hoc Power Analyses.**

**Supplementary item 1. Parameters and Results of Post-hoc Power Analyses.**

# post-hoc power analysis for one-way anova (age)

**One-way ANOVA to examine age differences**

***331 actual sample size***

*Computed using G*Power version 3.1.9.7*

**F tests -** ANOVA: Fixed effects, omnibus, one-way

**Analysis:** Post hoc: Compute achieved power

**Input:** Effect size f = 0.25

α err prob = 0.05

Total sample size = 331

Number of groups = 4

**Output:** Noncentrality parameter λ = 20.6875000

Critical F = 2.6322253

Numerator df = 3

Denominator df = 327

Power (1-β err prob) = 0.9777061

# post-hoc power analysis for independent t-test (Gender)

**Independent t-test to examine gender differences**

***330 actual sample size***

*Computed using G*Power version 3.1.9.7*

**t tests -** Means: Difference between two independent means (two groups)

**Analysis:** Post hoc: Compute achieved power

**Input:** Tail(s) = Two

Effect size d = 0.36

α err prob = 0.05

Sample size group 1 = 238

Sample size group 2 = 92

**Output:** Noncentrality parameter δ = 2.9324318

Critical t = 1.9672228

Df = 328

Power (1-β err prob) = 0.8324453

# post-hoc power analysis for Pearson correlation

**Pearson correlation to examine between-network construct validity of the Physician Well-Being Index-Expanded (ePWBI) and World Health Organization Well-Being Index (WHO-5)**

***333 actual sample size***

*Computed using G*Power version 3.1.9.7*

**Exact -** Correlation: Bivariate normal model

**Options:** exact distribution

**Analysis:** Post hoc: Compute achieved power

**Input:** Tail(s) = Two

Correlation ρ H1 = -0.543

α err prob = 0.05

Total sample size = 333

Correlation ρ H0 = 0

**Output:** Lower critical r = -0.1074982

Upper critical r = 0.1074982

Power (1-β err prob) = 0.9999999

# post-hoc power analysis for confirmatory factor analysis (CFA)

**Confirmatory factor analysis (CFA) for within-network construct validity of the ePWBI**

***333 actual sample size***

*Computed using “Webpower” Online Tool*

URL: <https://webpower.psychstat.org/models/sem01/>

**Power for SEM based on RMSEA**

| n | Rmsea0 | Rmsea1 | power | alpha |
| --- | --- | --- | --- | --- |
| 333 | 0.02 | 0.08 | 0.9961 | 0.05 |
